# The level of wasting and associated factors among children aged 6–59 months in sub-Saharan African countries: multilevel ordinal logistic regression analysis

**DOI:** 10.3389/fnut.2024.1336864

**Published:** 2024-06-06

**Authors:** Hiwot Altaye Asebe, Zufan Alamrie Asmare, Kusse Urmale Mare, Bizunesh Fantahun Kase, Tsion Mulat Tebeje, Yordanose Sisay Asgedom, Abdu Hailu Shibeshi, Afewerk Alemu Lombebo, Kebede Gemeda Sabo, Bezawit Melak Fente, Meklit Melaku Bezie, Beminate Lemma Seifu

**Affiliations:** ^1^Department of Public Health, College of Medicine and Health Sciences, Samara University, Samara, Ethiopia; ^2^Department of Ophthalmology, School of Medicine and Health Science, Debre Tabor University, Debre Tabor, Ethiopia; ^3^Department of Nursing, College of Medicine and Health Sciences, Samara University, Samara, Ethiopia; ^4^Department of Public Health, College of Medicine and Health Sciences, Samara University, Samara, Ethiopia; ^5^School of Public Health, College of Health Science and Medicine, Dilla University, Dilla, Ethiopia; ^6^Department of Epidemiology and Biostatics, College of Health Sciences and Medicine, Wolaita Sodo University, Sodo, Ethiopia; ^7^Department of Statistics, College of Natural and Computational Science, Samara University, Samara, Ethiopia; ^8^School of Medicine, College of Health Science and Medicine, Wolaita Sodo University, Sodo, Ethiopia; ^9^Department of Nursing, College of Medicine and Health Sciences, Samara University, Samara, Ethiopia; ^10^Department of General Midwifery, School of Midwifery, College of Medicine and Health Sciences, University of Gondar, Gondar, Ethiopia; ^11^Department of Public Health Officer, Institute of Public Health, College of Medicine and Health Sciences, University of Gondar, Gondar, Ethiopia; ^12^Department of Public Health, College of Medicine and Health Science, Samara University, Samara, Ethiopia

**Keywords:** wasting, 6–59 months children, SSA, multilevel ordinal logistic regression analysis, DHS

## Abstract

**Background:**

Despite various interventions to combat child malnutrition in sub-Saharan Africa, wasting remains a critical public health concern for children aged 6–59 months. Wasting is a significant predictor of child survival and development, with a heightened risk of mortality among children. However, there is a lack of recent comprehensive data on the prevalence, severity level, and factors contributing to wasting in this age group.

**Objective:**

To identify the severity levels of wasting and its individual and community-level factors contributing to wasting among children aged 6–59 months in Sub-Saharan African countries.

**Methods:**

This research utilized Demographic and Health Survey data from 34 Sub-Saharan African countries, spanning the period from 2007 to 2022. The study included a weighted sample of 180,317 6–59-month-old children. We employed a multilevel proportional odds model to identify factors predicting the severity of wasting. Adjusted odds ratios and 95% confidence intervals were reported to demonstrate significant relationships (*p* < 0.05) in the final model.

**Results:**

In Sub-Saharan Africa, 7.09% of children aged 6–59 months experience wasting (95% CI: 6.97, 7.20%). Among these children, the prevalence of moderate wasting is 4.97% (95% CI: 4.90, 5.10%), while severe wasting affects 2.12% (95% CI: 2.0, 2.20%). Factors such as term/post-term babies, wealth, frequency of feeding, improved toilet facilities, water sources, employed and educated mothers, rural residence, high community maternal education, and community media exposure are strongly associated with a lower chance of experiencing severe form of wasting. Conversely, birth order, family size, breastfeeding, diarrhea, cough, and fever, high community poverty, female household heads, and all Sub-Saharan Africa regions are linked to higher levels of wasting.

**Conclusion:**

The study findings underscore the persistent challenge of wasting among Sub-Saharan Africa’s children, with 7.09% affected, of which 4.97% experience moderate wasting and 2.12% severe wasting. The identified predictors of wasting highlight the complex interplay of socio-economic, environmental, and health-related determinants. To address this issue improve access to healthcare and nutrition services, enhance sanitation infrastructure, promote women’s empowerment, and implement community-based education programs. Additionally, prioritize early detection through routine screening and strengthen health systems’ capacity to provide timely interventions.

## Background

Wasting in under-five children is a serious global health issue (1). The World Health Organization (WHO) defines wasting as low weight-for-height or less than −2 SD (1). This typically signifies a recent and drastic reduction in weight, although it can also be a long-lasting condition.

Wasting in children is a critical health issue that arises from inadequate nutrition or repeated illnesses. It weakens the immune system, increases the risk of mortality, and can lead to long-term developmental issues, especially when it’s severe (2, 3). In 2022, 45 million children under the age of 5, which is 6.8% of this age group, were affected by wasting. Out of these, 13.7 million (2.1%) were experiencing severe wasting (4). More than three-quarters of all children with severe wasting live in Asia and another 22 percent live in Africa (5).

Though various strategies have been implemented to combat child malnutrition in Sub-Saharan Africa, wasting remains critically prevalent. This persistent high rate of malnutrition continues to be a major obstacle to achieving better health outcomes for children in the region (6, 7). In sub-Saharan Africa in 2017, 7.1% of children under five were wasted, or about 13.8 million children. Of these, 4 million were severely wasted (8). Furthermore, an estimated 6.3 million children under 5 in six countries in Africa’s Sahel region will be malnourished this year (9). In the East and Southern African region, over 1.5 million children are lacking critical treatment for severe malnutrition, which could save their lives (10). Childhood wasting has severe consequences, including increased mortality, susceptibility to illness, developmental delays, and long-term impacts on the economic and social potential and progress of the countries (11, 12). In countries where child mortality rates are high, being underweight contributes to approximately 15% of the overall burden on disability-adjusted life years (DALY) (13).

Previous studies revealed that inadequate energy and nutrient intake, nutrient loss due to infection, or a combination of both low energy or protein intake and high nutrient loss, maternal characteristics such as BMI, and education, as well as socio-economic condition and availability of services including water, sanitation, and hygiene, family size, and parental occupation were significant factors associated with wasting (14–19).

Several studies have proven that wasting among 6–59-month-old children is a serious public health problem in SSA. However, there is a paucity of recent evidence on the prevalence, severity level, and predictors of wasting in this age group in SSA. In addition, even though wasting has an ordinal nature, previous studies treat wasting as a binary outcome (have wasting vs. have no wasting). Therefore, simply classifying waste as either present or absent can miss important details and is not helpful for those who need to make choices or develop public health programs. Thus, this study identified both the individual and community-level predictors of wasting among children aged 6–59 months in SSA using a multilevel ordinal logistic regression model. Knowing the nuanced severity levels of wasting enables policymakers to refine strategies, enhancing public health programming and decision-making across the region.

## Methods data source and sampling procedure

We used the 2007 to 2022 DHS data from 34 Sub-Saharan African countries. The DHS is a survey conducted every 5 years to provide updated health and related indicators. The study subjects were chosen using a two-stage stratified sampling method. The first stage involved randomly selecting enumeration areas (EAs). The second step involved selecting households from the EAs. Each country’s survey comprises various datasets, such as those for men, women, children, births, and households. We used the Kids Record (KR) file. We selected relevant data from the KR dataset based on previous research. We then combined this data with another dataset using the STATA command “append using.” The total weighted sample size in this study was 180,317.

## Study variables and measurements

### Dependent variable

Our dependent variable was severity levels of wasting among 6–59 months children, We classified it into three ordinal categories.; (ordinal variable, which was categorized as severely wasted if a child’s WHZ score was below −3 SD, moderately wasted (− 3 ≤ WHZ < − 2), and not wasted WHZ ≥ − 2 SD).

### Independent variables

The predictor variables were based on two sources: individual-level and community-level factors.

Household factors: wealth index, sex of household head, family size, number of under-five children in the household, visit to a health facility, source of drinking water, and type of toilet facility (16, 20).

Maternal characteristics: maternal education and maternal working status, maternal media exposure (16, 21, 22).

Child characteristics: age of child, sex of child, twin status, order of birth, duration of pregnancy (preterm, term, post-term), breastfeeding status, fever, diarrhea, and cough within the past 2 weeks, and number of times a child feeds (23–26).

Community-level factors: a place of residence, SSA region, community poverty, media exposure, and maternal literacy.

Media exposure was measured by adding up three variables: watching television, listening to the radio, and reading newspapers. Mothers who were exposed to at least one of the three media sources listed were categorized as having media exposure. Mothers who were not exposed to any of the three media sources were categorized as not having media exposure. Three community-level variables were created: community maternal education, community media exposure, and community poverty. We derived these by aggregating individual-level data on maternal education, media exposure, and wealth status within each cluster or enumeration area. These variables were then classified as higher community maternal education, media exposure, and poverty based on the national median value of the proportion of maternal education and poverty. This classification was done since the distribution of these variables was not normal.

#### Operational definitions

Protected drinking water sources: include piped water, public taps, standpipes, tube wells, boreholes, protected dug wells and springs, rainwater, and bottled water (27).

Unprotected drinking water sources: include unprotected wells and springs, surface water (river, dam, lake, pond, stream, canal, irrigation channel), tanker trucks, carts with small tanks, and others (27).

Improved toilet facilities: include flush/pour flush toilets to piped water systems, septic tanks, and pit latrines; ventilated improved pit (VIP) latrines, pit latrines with slabs, and composting toilets (27).

Unimproved toilet facilities: include flush to somewhere else, flush do not know where, pit latrine without a slab/open pit, no facility/bush/field, bucket toilet, hanging toilet/latrine, and others (27).

### Data management and analysis

The statistical software STATA, version 17, was employed for data management and analysis. The WHO Anthro software was employed to generate WHZ scores. Descriptive results were presented using frequencies and percentages. Wasting, being a polychotomous variable with an ordinal nature, underwent analysis through an ordinal logistic regression model. Several types of ordinal logistic regression models were considered, such as the proportional odds model, partial proportional odds model with restrictions, partial proportional odds model without restrictions, continuous ratio model, and stereotype model. The proportional odds model, often utilized in epidemiological studies, was selected due to its frequent application.

This model assumes that the relationship between independent variables and the outcome variable is proportional; meaning the effect of each independent variable on the outcome is consistent across categories. The Brant test was conducted to assess the proportional odds assumption. Results indicated that the assumption was met (*p* > 0.05). Since the DHS data had a hierarchical structure, with children and mothers grouped within clusters, it was crucial to consider the variation between these clusters. To address this, a multilevel proportional odds model was used.

We calculated Likelihood Ratio (LR) tests, Variance Partition Coefficients (VPCs), and Median Odds Ratios (MORs) to figure out how much wasting changed between different clusters.

The VPC tells us how much of the total variation in wasting can be explained by the differences between the clusters, compared to the variation within each cluster. (28). The VPC was calculated using the formula.


$$VPC=6^{2}/\left(6^{2}+\pi^{2}/3\right)$$

Where π^2^/3 represents the variance of the standard logit distribution and σ_μ_^2^ is the cluster variance.

The MOR measures the heterogeneity in wasting among clusters on the odds ratio scale. It represents the median odds ratio between the cluster with a high likelihood of wasting and the cluster at lower risk when individuals are randomly selected from two clusters (EAs) (29).


$$\mathrm{MOR}=\exp.\surd \left(2*\partial^{2}*0.6745\right),\mathrm{MOR}=\exp0.95*\partial$$

In the multilevel logistic regression analysis, four distinct models were developed. The initial model, known as the null model, did not include any explanatory variables and was used to gauge the degree of cluster variation in wasting. The second model incorporated variables at the individual level, while the third model was adjusted for variables at the community level. The fourth model was a comprehensive one, integrating both individual and community-level variables. We compared the models using their deviance, represented by the -2Log-Likelihood Ratio (LLR). The model with the smallest deviance was deemed to be the most suitable for the data.

In the null model, unobserved factors at the community level accounted for approximately 1.05% of the total variation in wasting (VPC = 1.05%). This finding did not necessarily favor a multilevel ordinal model over a single-level one. However, further examination with the LR test, it was revealed that the multilevel ordinal logistic regression model outperformed the single-level ordinal logistic regression analysis. Additionally, the MOR suggested that transferring children from clusters with lower likelihoods of higher wasting levels to clusters with higher likelihoods could lead to 1.2 times higher levels of wasting. Therefore, the LR test and MOR supported the use of a multilevel ordinal logistic regression model.

Variables with a *p*-value of<0.25 in the bivariable multilevel proportional odds model were included for consideration in the multivariable multilevel proportional odds model. In the multivariable model, the strength of association was represented by the AOR with a 95% CI, and statistical significance was determined at *p* < 0.05.

## Ethical consideration

The current study utilized publicly available survey data from the MEASURE DHS program, eliminating the requirement for ethical review and informed consent. Permission to access and utilize the dataset for this research was obtained from http://www.dhsprogram.com. The datasets used do not contain any personal identifiers such as names or household addresses.

## Results

### Characteristics of the individual study participants

The study encompassed a total of 180,317 children between the ages of 6 and 59 months. Out of the total, 90,978 (50.45%) were males. About 39.8% of mothers did not receive any formal education. Around 21.85% of the children experienced fever, 21.25% had a cough, and 15.93% reported diarrhea within 2 weeks. Over half of the mothers (54.37%) were not exposed to any media, and the vast majority (83.45%) of the children were term babies (Table 1).

**Table 1:** Characteristics of the individual study participants (*n* = 180,317).

Variables	Categories	Weighted frequency	Percentage (%)
Child age (in months)	6-11 month	22,593	12.53
	12–23 month	42,091	23.34
	24-35 months	39,301	21.80
	36-47 months	39,022	21.64
	48-59 months	37,310	20.69
Child sex	Female	89,339	49.55
	Male	90,978	50.45
Child twin status	Single	174,677	96.87
	Multiple	5,640	3.13
Duration of pregnancy	Preterm	20,578	11.41
	Term	150,472	83.45
	Post-term	9,267	5.14
Duration of breastfeeding	still breastfeeding	55,069	30.54
	never breastfed	15,466	8.58
	ever breastfed, not now	109,781	60.88
Birth order	1st	37,820	20.97
	2nd or 3rd	63,423	35.17
	4th or 5th	41,722	23.14
	6th or above	37,352	20.71
Household wealth index	Poor	88,980	49.35
	Middle	34,323	19.03
	Rich	57,014	31.62
Maternal educational status	No education	71,763	39.80
	Primary	59,882	33.21
	Secondary	41,566	23.05
	Higher	7,106	3.94
Maternal working status	Not employed	77,140	42.78
	Employed	103,177	57.22
Family size	1 to 3	17,441	9.67
	4 to 6	80,722	44.77
	7 and above	82,154	45.56
Media exposure	Yes	82,279	45.63
	No	98,038	54.37
Sex of household head	Male	139,357	77.28
	Female	40,960	22.72
Source of drinking water	Protected	114,532	63.52
	Unprotected	65,785	36.48
type of toilet facility	Improved	79,883	44.30
	Not improved	100,434	55.70
Had a fever in the last 2 weeks	Yes	39,395	21.85
	No	140,922	78.15
Had diarrhea in the last 2 weeks	Yes	28,731	15.93
	No	151,586	84.07
Had a cough in the last 2 weeks	Yes	38,314	21.25
	No	142,003	78.75

### Community-level features of participants

Among the study participants 41.2% were from West Africa, and 35.61% were from East Africa More than two-thirds (68.96%) of participants were rural residents. Concerning community maternal literacy, media exposure, and poverty, about 95,993 (53.24%), 83,406(46.26%), and 90,917(50.42%) of the participants were from the community with the highest levels of maternal literacy, media exposure, and poverty, respectively (Table 2).

**Table 2:** Community-level characteristics of the respondents in sub-Saharan Africa (*n* = 180,317).

Variables	Categories	Weighted frequency	Percentage (%)
Residence	Urban	55,963	31.04
	Rural	124,354	68.96
Community-maternal education	Low	84,324	46.76
	High	95,993	53.24
Community poverty	Low	89,400	49.58
	High	90,917	50.42
Community media exposure	Low	96,911	53.74
	High	83,406	46.26
Region	Central Africa	35,255	19.55
	Eastern Africa	64,207	35.61
	Western Africa	74,295	41.20
	Southern Africa	6,560	3.64

### Prevalence of wasting among children aged 6–59 months in SSA

7.09% of children 6–59 months in SSA are wasted (95% CI: 6.97, 7.20%). The percentage of wasted children varies from 4.30% in Southern Africa to 7.1% in West Africa. By the severity of wasting, the prevalence of moderate and severe wasting among children of the 6–59 month age group in Sub-Saharan Africa were 4.97% (95% CI: 4.90, 5.10%) and 2.12% (95% CI: 2.0, 2.20%), respectively (Table 3).

**Table 3:** Prevalence of wasting among 6–59 months children in sub-Saharan African countries evidence from DHS data (*n* = 180,317).

Countries	DHS year	Total weighted sample population	Wasting		
			Normal (%)	Moderate (%)	Severe (%)
Angola	2015/16	5,727	5,323 (92.95)	275 (4.80)	129 (2.25)
Burkinafaso	2021	5,167	4,814 (93.17)	235 (4.55)	118 (2.28)
Benin	2017/18	10,687	9,926 (92.88)	506 (4.73)	255 (2.39)
Burundi	2016	5,427	5,046 (92.98)	256 (4.72)	125 (2.30)
Democratic Republic of Congo	2013/2014	7,367	6,844 (92.90)	360 (4.89)	163 (2.21)
Republic of Congo	2011/12	4,020	3,722 (92.59)	206 (5.12)	92 (2.29)
Cotedivoire	2021	4,251	3,938 (92.64)	221 (5.2)	92 (2.16)
Cameroon	2018	3,998	3,688 (92.24)	209 (5.23)	101 (2.53)
Ethiopia	2016	8,056	7,558 (93.82)	346 (4.29)	152 (1.89)
Gabon	2019/21	4,755	4,440 (93.37)	222 (4.70)	93 (1.93)
Ghana	2014	2,413	2,267 (93.94)	103 (4.30)	43 (1.76)
Gambia	2019/20	3,273	3,025 (92.42)	169 (5.16)	79 (2.42)
Guinea	2018	3,082	2,882 (93.51)	148 (4.80)	52 (1.69)
Kenya	2022	15,441	14,281 (92.50)	785 (5.10)	375 (2.40)
Comoros	2012	2,401	2,237 (93.16)	109 (4.54)	55 (2.30)
Liberia	2019/20	2,181	2,005 (91.93)	111 (5.10)	65 (2.97)
Lesotho	2014	1,163	1,062 (91.32)	69 (5.93)	32 (2.75)
Madagascar	2021	5,136	4,724 (92.00)	291 (5.70)	121 (2.3)
Mauritania	2019/21	8,744	8,071 (92.30)	469 (5.40)	204 (2.30)
Malawi	2015/2016	4,715	4,317 (91.60)	268 (5.70)	130 (2.70)
Mozambique	2011	8,643	8,072 (93.40)	404 (4.70)	167 (1.9)
Nigeria	2012	7,957	7,415 (93.19)	353 (4.44)	189 (2.37)
Niger	2012	4,532	4,188 (92.41)	238 (5.25)	106 (2.34)
Rwanda	2019/20	3,435	3,194 (93.00)	160 (4.70)	81 (2.30)
Sierra Leone	2019	3,646	3,394 (93.10)	191 (5.24)	61 (1.66)
Senegal	2019	4,914	4,571 (93.00)	240 (4.90)	103 (2.1)
Eswatini	2006/2007	1,877	1,741 (92.75)	94 (5.00)	42 (2.25)
Chad	2014/15	9,244	8,581 (92.83)	460 (4.98)	203 (2.19)
Togo	2013/14	2,928	2,721 (92.93)	146 (4.99)	61 (2.08)
Tanzania	2015/16	8,004	7,358 (91.93)	478 (5.97)	168 (2.10)
Uganda	2016	3,933	3,602 (91.58)	261 (6.63)	70 (1.79)
South Africa	2016	988	924 (93.52)	50 (5.06)	14 (1.42)
Zambia	2018/19	7,803	7,305 (93.62)	432 (5.54)	66 (0.84)
Zimbabwe	2015	4,409	4,294 (97.39)	105 (2.38)	10 (0.23)
Regions					
Central Africa		35,255	32,737 (92.86)	1736 (4.92)	782 (2.22)
Eastern Africa		64,207	59,483 (92.64)	3,382 (5.27)	1,342 (2.09)
Western Africa		74,295	69,030 (92.91)	3,628 (4.88)	1,637 (2.21)
Southern Africa		6,560	6,280 (95.73)	224 (3.41)	56 (0.86)

*Numbers outside parentheses represent total weighted sample populations.

### Multivariable multilevel proportional odds model analysis results

#### Individual and community-level factors associated with wasting

Being born term/post-term, family wealth, frequency of child feeding, improved toilet facilities, improved water sources, health facility visit, employed mother, rural residence, formal maternal education, media exposure, high community maternal education, high community media exposure were strongly linked to a lower chance of having a more severe form of wasting. In contrast, higher birth order, family size, breastfeeding status, diarrhea, cough, and fever in the past 2 weeks, high community poverty, female household heads, and all sub-Saharan Africa regions were strongly linked with higher levels of wasting.

The likelihood of having higher levels of wasting among children who feed 3to4 times a day decreased by 37% (AOR = 0.63, 95% CI: 0.59–0.66) compared to children who did not get food. The odds of having higher levels of wasting have decreased by 8% (AOR = 0.92, 95% CI: 0.87–0.98) for children born at term compared to preterm children. Children of mothers with primary and secondary education were at reduced risk of having severe wasting by 33 and 23%, respectively, than children of mothers without formal education. Children who never breastfed had1.72 times (AOR = 1.17, 95% CI: 1.6–1.84) times higher odds of having higher levels of wasting compared to those who still breastfed.

Children born sixth or later were 1.15 times (AOR = 1.15, 95% CI: 1.08–1.23) more likely to experience higher levels of wasting than first-born children. The likelihood of children experiencing higher levels of wasting was reduced by 53% (AOR = 0.47, 95% CI: 0.44–0.51) for those from middle-income households and by 51% (AOR = 0.49, 95% CI: 0.46–0.53) for those from rich households when compared to children from low-income households.

Children who had experienced diarrhea in the past 2 weeks were 1.53 times more likely (AOR = 1.53, 95% CI: 1.45–1.60) to exhibit higher levels of wasting compared to children who had not experienced diarrhea.

Children in East and West African regions had 1.42 times (AOR = 1.42, 95% CI: 1.25–1.61) and 1.19 times (AOR = 1.19, 95% CI: 1.04–1.36) higher odds of higher levels of wasting than those in South Africa.

A 16%(AOR = 0.84, 95% CI: 0.80–0.88) reduction in the odds of higher levels of wasting is observed among children who live in communities with higher levels of maternal education, compared to children who live in communities with lower levels of maternal education (Tables 4, 5).

**Table 4:** Bivariable multilevel proportional ordinal logistic regression analysis of individual and community level variables associated with levels of wasting among children aged 6–59 month in sub-Saharan African countries.

Variables	Categories	Crude odds ratio (95%CI)	*P*-value
Child age (in months)	6–11 month	1	
	12–23 month	1.00 (0.94,1.06)	0.996
	24–35 months	1.00 (0.94,1.07)	0.976
	36–47 months	0.96 (0.90,1.03)	0.259
	48–59 months	0.99 (0.92, 1.05)	0.763
Child sex	Male		
	Female	0.99 (0.96,1.02)	0.622
Child twin status	Single	1	
	Multiple	0.97 (0.87, 1.07)	0.598
Duration of pregnancy	Preterm	1	
	Term	0.90 (0.85,0.95)	0.001
	Post term	0.86 (0.78,0.95)	0.003
Duration of breast feeding	still breastfeeding	1	
	never breastfed	1.68 (1.58,1.79)	0.001
	ever breastfed, not now	0.98 (0.94,1.02)	0.287
Birth order	1st	1	
	2nd or 3rd	1.05 (1.004,1.11)	0.035
	4th or 5th	1.18 (1.12,1.25)	0.001
	6th or above	1.48 (1.39,1.56)	0.001
Household wealth index	Poor	1	
	Middle	0.39 (0.37, 0 0.41)	0.001
	Rich	0.39 (0.37,0.41)	0.001
Maternal educational status	No education	1	
	Primary	0.56 (0.54,0.59)	0.001
	Secondary	0.49 (0.47,0.52)	0.001
	Higher	0.50 (0.45,0.56)	0.001
Maternal working status	Not employed	1	
	Employed	0.85 (0.82,0.88)	0.001
Family size	1 to 3	1	
	4 to 6	1.17 (1.09,1.24)	0.001
	7 and above	1.31 (1.22, 1.39)	0.001
Media exposure	No		
	Yes	0.59 (0.57, 0.62)	0.001
Sex of household head	Male	1	
	Female	1.022 (0.98, 1.07)	0.310
Source of drinking water	Protected	1	
	Unprotected	1.42 (1.37,1.47)	0.001
type of toilet facility	Improved	1	
	Not improved	1.77 (1.70,1.84)	0.001
Had a fever in the last 2 weeks	No	1	
	Yes	1.54 (1.48,1.60)	0.001
Had diarrhea in the last 2 weeks	No	1	
	Yes	1.73 (1.66,1.81)	0.001
Had cough in the last 2 weeks	No	1	
	Yes	1.38 (1.32,1.43)	0.001
Residence	Urban	1	
	Rural	1.14 (1.09, 1.18)	0.001
Community-maternal education	Low	1	
	High	0.55 (0.53, 0.57)	0.001
Community poverty	Low	1	
	High	1.99 (1.92,2.06)	0.001
Community media exposure	Low	1	
	High	0.54 (0.52, 0.56)	0.001
Region	Southern Africa	1	
	Eastern Africa	1.79 (1.58, 2.02)	0.001
	Western Africa	1.72 (1.52,1.94)	0.001
	Central Africa	1.74 (1.53,1.97)	0.001

**Table 5:** Multilevel ordinal logistic regression analysis of individual and community level variables associated with severity levels of wasting among children aged 6–59 months in sub-Saharan African countries.

Variables	Null model	Model I	Model II	Model III
**Number of under-five children**				
Only one		1		1
morethan1child		1.08 (1.02, 1.13)		1.079 (1.03, 1.13)*
**Family size**				
1to3		1		1
4 to6		1.08 (0.99, 1.16)		1.084 (1.001, 1.17)*
> = 7		1.09 (1.002, 1.8)		1.097 (1.009, 1.19)*
**Birth order**				
1st		1		1
2nd or 3rd		0.99 (0.94, 1.05)		0.99 (0.93, 1.05)
4th or 5th		1.01 (0.95, 1.08)		1.01 (0.96, 1.08)
6th or above		1.16 (1.08, 1.23)		1.15 (1.083, 1.23)*
**Media exposure**				
No		1		1
Yes		0.81 (0.78, 0.85)		0.88 (0.84, 0.92)*
**Maternal**				
No educational		1		1
Primary		0.64 (0.61, 0.67)		0.67 (0.64, 0.71)*
Secondary		0.72 (0.68, 0.77)		0.77 (0.72, 0.82)*
Higher		0.93 (0.81, 1.05)		0.91 (0.79, 1.04)
**Toilet facility**				
Not improved		1		1
Improved		0.80 (0.77, 0.84)		0.77 (0.73, 0.80)*
**Breastfeeding**				
Still breastfeeding		1		1
Never breastfed		1.86 (1.74, 1.99)		1.72 (1.61, 1.83)*
Ever breastfed, not now		1.13 (1.08, 1.18)		1.13 (1.08, 1.18)*
**Wealth status**				
Poor		1		1
Middle		0.44 (0.42, 0.47)		0.48 (0.44, 0.50)*
Rich		0.51 (0.48, 0.53)		0.49 (0.46, 0.53)*
**Frequency of feeding**				
None		1		1
1to 2times		0.84 (0.79, 0.89)		0.86 (0.81, 0.91)*
3 to4times		0.62 (0.59, 0.66)		0.63 (0.59, 0.66)*
5andabove times		0.64 (0.59, 0.67)		0.64 (0.60, 0.68)*
**Maternal working status**				
Not employed		1		1
Employed		0.86 (0.83, 0.89)		0.88 (0.85, 0.92)*
**The health facility in the past 12 months**				
No		1		1
Yes		0.93 (0.89, 0.97)		0.93 (0.89, 0.96)*
**Diarrhea in the past 2 weeks**				
No		1		1
Yes		1.54 (1.47, 1.62)		1.53 (1.45, 1.60)*
**Fever in the past 2 weeks**				
No		1		1
Yes		1.23 (1.17, 1.28)		1.29 (1.23, 1.36)*
**Cough in past 2 weeks**				
No		1		1
Yes		1.29 (1.23, 1.35)		1.29 (1.23, 1.36)*
**Sex of household head**				
Male		1		1
Female		1.07 (1.02, 1.12)		1.06 (1.01, 1.11)*
**Source of drinking water**				
Un-protected		1		
Protected		0.94 (0.91, 0.98)		0.92 (0.89, 0.96)*
**Duration of pregnancy**				
Preterm		1		1
Term		0.89 (0.84, 0.94)		0.92 (0.87, 0.98)*
Post-term		0.82 (0.75, 0.91)		0.86 (0.78, 0.96)*
**Place of residence**				
Urban			1	1
Rural			0.59 (0.56, 0.62)	0.52 (0.49, 0.54)*
**Community media exposure**				
Low			1	1
High			0.64 (0.62, 0.7)	0.76 (0.72, 0.79)*
**Community maternal education**				
Low			1	1
High			0.67 (0.65, 0.70)	0.84 (0.80, 0.88)*
**Community poverty**				
Low			1	1
High			1.93 (1.84, 2.02)	1.21 (1.15, 1.28)*
**Region**				
Southern Africa			1	1
Central Africa			1.41 (1.24, 1.61)	1.14 (1.001, 1.30)*
Eastern Africa			1.56 (1.37, 1.76)	1.42 (1.25, 1.62)*
Western Africa			1.48 (1.30, 1.67)	1.19 (1.04, 1.36)*
/cut1	13.23 (12.94, 13.53)	1.84 (1.72, 1.94)	2.61 (2.48, 2.75)	1.59 (1.40, 1.78)*
/cut2	3.84 (3.81, 3.88)	3.13 (3.02, 3.25)	3.89 (3.75, 4.03)	2.89 (2.71, 3.08)*
**Random effect analysis result**				
Variance	0.035	0.046	0.056	0.05
VPC (%)	1.05	1.38	1.67	1.50
MOR	1.20	1.23	1.25	1.24
LR test	Prob > = chibar2 = 0.00001			
LLR	−53920.7	−48900.45	−52643.58	−48516.73
Deviance	107841.4	97800.9	105287.16	97033.46
AIC	107847.4	97860.89	105307.2	97107.46
BIC	107877.7	98162.59	105408.2	97479.56

AIC, akaike information criteria; BIC, bayesian information criteria; LLR, log-likelihood ratio; LR, likelihood ratio; MOR, median odds ratio; VPC, variance partition coefficient.* indicates significant variables.

## Discussion

The primary objective of this study was to delve into the severity levels of wasting and pinpoint both individual and community-level factors that contribute to wasting among children aged 6–59 months in Sub-Saharan African countries. This aim stems from the pressing need to address the critical issue of child malnutrition, particularly wasting, which poses a significant threat to child health and survival in these regions.

Our findings reveal a diverse range of severity levels of wasting, highlighting the complexity of the issue at hand. The factors contributing to these levels are multifaceted, encompassing both individual and community-level elements. This underscores the necessity for a comprehensive approach to tackling wasting, one that goes beyond addressing individual nutritional needs and includes broader community and societal interventions their own nutritional challenges. Empowering communities to address their own nutritional challenges is paramount for effective intervention strategies in combating wasting among children in Sub-Saharan Africa (30). By involving local stakeholders in identifying priorities, designing solutions, and implementing programs tailored to their specific needs and contexts, a participatory approach fosters ownership and ensures the sustainability of interventions (6). Furthermore, integrating individual and community-level interventions can yield synergistic effects, amplifying the impact on reducing wasting and improving child health outcomes. Community-level interventions complement individual efforts by creating supportive environments that reinforce healthy behaviors and access to resources (31, 32). Recognizing the significance of community-level factors, policy changes must prioritize these interventions in health programming and decision-making processes. This may necessitate reallocating resources, forging stronger partnerships with community-based organizations, and incorporating community perspectives into policy development. Such measures are essential for addressing wasting effectively and promoting child health across Sub-Saharan Africa.

In this study wasting, a serious form of malnutrition, affects 7.09% (95% CI, 6.9, 7.20%) of children aged 6–59 months in Sub-Saharan African countries (SSA), with prevalence varying across regions, indicating that wasting is a significant health issue in SSA. This higher than theglobal prevalence of wasting (6.8%) among under five children (33) and studies conducted in Brazil and China (34, 35). This is because children in SSA have been malnourished for a long time as a result of enduring poverty in the region (36). Additionally, the low economic status of many African countries can restrict people’s ability to obtain enough nutritious food, which can lead to wasting (37).

Children from households in the middle and upper wealth quintiles are less likely to experience severe wasting than children from households in the poor wealth quintile. This conclusion aligns with these studies (16, 20, 24, 38–42). The potential explanation for this is that insufficient financial resources often lead to limited access to nutritious food, as impoverished families may struggle to afford a balanced diet that provides essential vitamins and minerals. Additionally, impoverished living conditions may expose children to unsanitary environments and unsafe water sources, increasing the risk of infections that further exacerbate nutritional challenges. Limited access to healthcare and educational resources within impoverished communities can also hinder parents’ awareness of proper nutrition practices, perpetuating the cycle of poor dietary habits (43–45).

Children who had diarrhea, fever, and cough in the past 2 weeks were more likely to be wasted than children who did not have these illnesses (17, 46–48). This is because infections significantly compromise children’s nutritional status by disrupting their dietary intake, absorption, and metabolism. Illnesses such as fever, cough, and diarrhea increase the body’s metabolic demands, necessitating higher energy and nutrient intake. However, the accompanying symptoms, such as decreased appetite, difficulty breathing, and gastrointestinal disturbances, often lead to reduced food consumption and nutrient absorption. Diarrhea, in particular, exacerbates the situation by causing rapid loss of fluids and essential nutrients. Moreover, the cyclical relationship between malnutrition and susceptibility to infections perpetuates a vicious cycle that poses long-term threats to a child’s overall health and well-being (49).

Children who fed less than three times per day were more prone to being wasted than their counterparts. This finding was aligned with the findings from similar studies (17, 50, 51). This is because, when a child is not fed regularly, it often leads to a chronic energy deficit, where the calories consumed are insufficient to meet the energy expenditure required for growth and development. This imbalance can result in the body utilizing its stores for energy, leading to unintended weight loss and a higher risk of wasting. Inadequate intake of essential nutrients, such as proteins and micronutrients, due to infrequent meals, further exacerbates the problem, compromising the child’s ability to build and maintain tissues. Additionally, irregular feeding patterns can disrupt metabolic processes, impacting the body’s ability to utilize nutrients efficiently (52).

The current study found that children who are part of a family with more than four members are at greater risk of being wasted than those from families with fewer than four members. This result is consistent with research done in Vietnam and Bangladesh (53, 54). This is attributable to larger families may face more financial pressure when it comes to food consumption, which could potentially lead to poorer nutritional status among the children.

The present results showed that employed and educated mothers were less likely to have wasted children than those who are not employed and uneducated. Unemployed mothers may face challenges due to financial constraints, such as providing nutritious food at the required frequency, ensuring adequate care, and accessing health care services. This finding is in line with research done in Nigeria, Ethiopia, and Afganistan (55–57).

Children in rural areas are less likely to be malnourished than children in urban areas. This finding is consistent with previous studies (58–60) On the other hand, some studies have found the opposite (18, 61, 62) the reason behind this is, that malnutrition is more common in rural areas due to factors such as low education levels, low social status, poor water quality, and high rates of infectious disease. However, rapid urbanization and the resulting high levels of poverty and hunger are now thought to be the main causes of malnutrition in urban areas (63).

Protected water sources and improved toilet facilities reduce the risk of wasting among children. This finding agrees with this stud (64–65). This is because a child who lives in a community with improved sanitation is less likely to be exposed to fecal matter in the environment, which reduces the risk of diarrhea and other infections and they will have a good appetit (66).

Maternal media exposure reduces the level of wasting among their children compared to their counterparts. This finding corresponds to the study conducted in North Africa (66). This can be explained by, improved access to information empowering mothers to adopt healthier behaviors and adhere to recommended feeding practices, ultimately contributing to better child nutrition outcomes. Furthermore, media exposure can also influence social norms and community perceptions regarding nutrition, fostering an environment that supports and encourages proper child-feeding practices (70).

This study indicates that children from households headed by females are more likely to experience wasting compared to those from households headed by males. This is in line with the study conducted in Nepal (57). The justification behind this conclusion is that female heads of households often carry a double burden in caring for their dependents and being the sole breadwinner of the family. This can lead to stress and limited time for proper child care, including feeding practices (67).

### Strengths and limitations of this study

The findings of our study should be considered with certain restrictions. The cross-sectional design of the DHS data prevents us from determining cause-and-effect relationships. Furthermore, our study only accounted for children who were alive during the data collection period, omitting any fatalities that might have resulted from wasting-related complications (indicating survivor bias). However, despite these constraints, our study possesses several notable advantages. It utilizes a combined, nationally representative DHS survey encompassing 34 sub-Saharan African countries. Additionally, the substantial sample size of our study ensures adequate power to discern the genuine impact of the independent variables.

## Conclusion

The study findings underscore the persistent challenge of wasting among children aged 6–59 months in Sub-Saharan Africa, with 7.09% affected, of which 4.97% experience moderate wasting and 2.12% severe wasting. The identified factors associated with wasting highlight the complex interplay of socio-economic, environmental, and health-related determinants. To address this issue effectively, interventions should adopt a multifaceted approach that targets vulnerable populations, improves access to healthcare and nutrition services, enhances sanitation infrastructure, promotes women’s empowerment, and implements community-based education programs. Additionally, prioritizing early detection through routine screening and strengthening health systems’ capacity to provide timely interventions are crucial steps toward reducing the burden of wasting and improving child health outcomes in the region.

## Data availability statement

Publicly available datasets were analyzed in this study. This data can be found here: Data is available online and you can access at: www.measuredhs.com.

## Ethics statement

Ethical approval was not required for the studies involving humans because The current study utilized publicly available survey data from the MEASURE DHS program, eliminating the requirement for ethical review and informed consent. Permission to access and utilize the dataset for this research was obtained from http://www.dhsprogram.com. The datasets used do not contain any personal identifiers such as names or household addresses. The studies were conducted in accordance with the local legislation and institutional requirements. Written informed consent for participation was not required from the participants or the participants’ legal guardians/next of kin in accordance with the national legislation and institutional requirements because The current study utilized publicly available survey data from the MEASURE DHS program, eliminating the requirement for ethical review and informed consent. Permission to access and utilize the dataset for this research was obtained from http://www.dhsprogram.com. The datasets used do not contain any personal identifiers such as names or household addresses.

## Author contributions

HA: Conceptualization, Data curation, Formal analysis, Methodology, Software, Supervision, Writing – original draft, Writing – review & editing. ZA: Conceptualization, Data curation, Writing – original draft, Writing – review & editing. KM: Conceptualization, Formal analysis, Methodology, Writing – original draft. BK: Data curation, Methodology, Writing – original draft. TT: Conceptualization, Software, Writing – original draft. YA: Formal analysis, Writing – original draft. AH: Methodology, Software, Writing – review & editing. AL: Writing – original draft, Writing – review & editing. KS: Data curation, Writing – review & editing. BF: Data curation, Writing – original draft, Writing – review & editing. MB: Conceptualization, Data curation, Writing – original draft, Writing – review & editing. BS: Conceptualization, Data curation, Methodology, Software, Writing – original draft, Writing – review & editing.
